# Chronic rhinosinusitis with nasal polyps: Key considerations in the multidisciplinary team approach

**DOI:** 10.1002/clt2.70010

**Published:** 2025-01-10

**Authors:** Oliver Pfaar, Anju T. Peters, Camille Taillé, Thijs Teeling, Jared Silver, Robert Chan, Peter W. Hellings

**Affiliations:** ^1^ Department of Otorhinolaryngology, Head and Neck Surgery Section of Rhinology and Allergy University Hospital Marburg Philipps‐Universität Marburg Marburg Germany; ^2^ Allergy‐Immunology Division and the Sinus and Allergy Center Feinberg School of Medicine Northwestern University Chicago Illinois USA; ^3^ Reference Center for Rare Pulmonary Diseases and University of Paris Cité Inserm 1152 Hospital Bichat ‐ Claude‐Bernard Paris France; ^4^ Patient Advisor The Netherlands; ^5^ US Medical Affairs—Respiratory GSK Durham North Carolina USA; ^6^ Clinical Sciences, Respiratory GSK Brentford UK; ^7^ Department of ENT University of Leuven Leuven Belgium

**Keywords:** chronic rhinosinusitis, ENT diseases, multidisciplinary care team, nasal polyps, standard of care

## Abstract

**Background:**

Chronic rhinosinusitis with nasal polyps (CRSwNP) is a recurrent inflammatory disease associated with several comorbidities and a significant disease burden for patients. Treatments include corticosteroids and sinonasal surgery, but these can be associated with the risk of adverse events and nasal polyp recurrence. Biologic treatments such as mepolizumab can be used as an add‐on treatment and are effective at reducing surgery and corticosteroid use.

**Main text:**

Patients with CRSwNP may be seen by a specialist in one of several different areas and often experience delayed diagnosis due to the need to see multiple physicians, as well as misdiagnosis resulting from lack of sufficient expertise within any one speciality. Multidisciplinary team (MDT) approaches have been shown to be effective in optimising the treatment and clinical management of other respiratory diseases, such as aspirin‐exacerbated respiratory disease and severe asthma. In CRSwNP, an MDT approach may reduce diagnostic delays, mitigate secondary disease burden, and reduce overprescription of corticosteroids and antibiotics.

**Conclusion:**

This article provides an overview of the patient perspective of MDTs, existing approaches and barriers to adoption, lessons learnt from allied and rare diseases, how to address under‐recognised aspects of CRSwNP, and other key considerations for developing an MDT approach.

## 1 INTRODUCTION

Chronic rhinosinusitis with nasal polyps (CRSwNP) is a recurrent inflammatory disease.^1^, ^2^ Patients present with symptoms including nasal congestion, loss of smell, facial pressure or pain, and chronic rhinorrhoea; these symptoms occur in tandem with sinonasal inflammation and nasal polyps (NPs), which are measured by sinus computed tomography (CT) scan or nasal endoscopy.^3^ CRSwNP accounts for 25%–30% of all chronic rhinosinusitis (CRS) cases,^3^ with a prevalence of 0.5%–4.0% based on studies conducted using questionnaires and/or nasal endoscopy.^4^ CRS can be further subdivided into endotypes based on cellular and molecular factors, including the presence of eosinophils, cytokine profile, innate lymphoid cells, and T‐cell subsets; CRSwNP is predominantly associated with the type 2 inflammation endotype in Western populations.^5^, ^6^

Patients with CRSwNP often have comorbid asthma, and the disease is also associated with chronic obstructive pulmonary disease, aspirin‐exacerbated respiratory disease (AERD), hypogammaglobulinemia, and gastro‐oesophageal reflux disease.^7^, ^8^ Overall, CRSwNP is a burdensome disease and patients have significantly lower physical and mental health‐related quality of life (HRQoL) compared with population norms.^9^ In addition to the primary symptoms of CRSwNP, patients experience secondary burdens such as breathing difficulties, sleep impairment, mood disturbances, and impaired social functioning,^10^, ^11^, ^12^ which could also affect HRQoL.

Treatment for CRSwNP includes saline irrigations, sinus implants, corticosteroids, and/or sinonasal surgery.^7^, ^8^ The European Position Paper on Rhinosinusitis and Nasal Polyps (EPOS) and European Forum for Research and Education in Allergy and Airway Diseases (EUFOREA) guidelines recommend intranasal corticosteroids as first‐line therapy,^7^, ^8^ and real‐world data reflect this recommendation: an analysis of claims data in Germany indicated that intranasal corticosteroids are used as initial treatment for most patients with CRSwNP, and systemic corticosteroids are frequently prescribed for patients with severe disease.^13^ However, corticosteroids and surgery are associated with non‐negligible levels of risk as well as NP recurrence and the need for additional revision surgery.^9^ Additionally, it is well accepted that endoscopic sinus surgeries are unable to control underlying inflammation.^14^ Although oral corticosteroids (OCS) are effective in the treatment of nasal polyps, at present, there is no consensus regarding the indications, timing, dosage, time course, and safety of their administration. Therefore, evidence‐based guidelines for the use of OCS in patients with CRSwNP remain necessary.^14^

The decision to treat CRSwNP with additional corticosteroids or switch to surgery is influenced by many factors, including surgeon's preference, patient's desire, treatment availability, and comorbidities.^14^ The complexity of therapeutic choice in practice has been increased by the emergence of biologics such as dupilumab, mepolizumab, and omalizumab as add‐on maintenance treatments for patients with inadequately controlled CRSwNP.^15^, ^16^, ^17^, ^18^, ^19^, ^20^ In clinical trials, mepolizumab and dupilumab have been shown to reduce OCS use and the need for additional surgery compared with placebo.^9^, ^21^, ^22^

Patients with CRSwNP may be seen by a physician specialising in one of several different areas (i.e., allergy/immunology, pulmonology, ear nose and throat [ENT], internal medicine, primary medicine, or paediatrics), particularly if they have comorbidities. The need to see multiple physicians may lead to diagnostic delays, which contribute to additional disease impact.^23^ Misdiagnosis can also be a delaying factor, with common misdiagnoses including cold/viral infections, allergic rhinitis, sinusitis, and migraine.^24^, ^25^ These diagnoses are often treated with antibiotics and corticosteroids,^26^ which may be unnecessary in some cases. A multidisciplinary team (MDT) approach involving physicians from multiple specialities in a patient's treatment plan could optimise the treatment and clinical management of CRSwNP,^3^, ^27^ for example, by reducing diagnostic delays, mitigating secondary disease burdens, and reducing overprescription of corticosteroids and antibiotics. Additionally, this approach can also play an important role in selecting the appropriate surgical intervention for patients and deciding if an expanded‐function or limited‐function endoscopy would be most beneficial.^28^ It is possible that by encouraging early intervention (medical or surgical) and improving long‐term symptomatic management, MDTs could improve disease trajectory over time, although appropriately powered trials are needed as confirmation. The MDT approach may be particularly important for patients with comorbidities who may be more likely to experience overprescription of corticosteroids^29^; in the recent EUFOREA pocket guide for CRS, recommendations for these patients include timely referral to specialists and an MDT approach.^8^ Patients with type 2 inflammation are predisposed to several comorbidities (e.g. asthma, eosinophilic oesophagitis, and atopic dermatitis), underlining the importance of an interdisciplinary approach for optimal patient care.^30^ Even where appropriate treatment options are available, disease management and patient outcomes can be negatively impacted if the MDT approach is suboptimal.^27^, ^31^ In addition to the direct benefit to a particular patient's treatment plan, being part of an MDT facilitates the development of cross‐disciplinary skills for healthcare providers (HCPs); for example, allergists/immunologists can provide guidance on testing, interpretation, and management of comorbid conditions such as asthma.^3^, ^27^ Adoption of an MDT approach has led to improved outcomes in chronic respiratory diseases, including severe asthma.^32^, ^33^, ^34^

This review article aims to provide insights and discussion of the benefits of an MDT approach for managing CRSwNP based on published literature, clinical experience and the patient perspective (including an illustrative case study). Current barriers to MDTs becoming the standard of care are also considered.

## 2 A PATIENT PERSPECTIVE ON THE BENEFITS OF MDT APPROACHES IN CRSwNP TREATMENT

### 2.1 CRSwNP case example

In the patient author's (TT's) experience, communication with his physicians has improved over time with increasing personal experience of CRSwNP, but this contrasts with the experience of many other patients with the disease. There were initial delays in diagnosis, with nasal problems since childhood not recognised as an issue by physicians. No CT scan was carried out until after the initial diagnosis of severe asthma (partially due to technology availability), and a diagnosis of NP was not made until the patient was ∼50 years old after a severe asthma attack. This led to delays in treatment. The patient's first of five sinus surgeries (septoplasty) was at 21 years of age; after the second surgery, the treating physician concluded that the patient had an allergic condition.

The patient is involved in decision‐making related to treatment, and treatment ownership has evolved through experience over the years, including as a member of a number of European patient advisory boards. However, from speaking to many others, the patient estimates that 50% of other patients have no ownership of their treatment plan, 25%–30% have some ownership, and 10% are knowledgeable and involved. Educating patients on how to prepare for their annual physician appointment may help to improve ownership, knowledge and involvement in treatment decisions.

### 2.2 The patient experience of an MDT approach

Initially, the MDT approach was not typically used owing to issues with lack of familiarity/understanding and competition between respiratory and ENT specialist teams, and even now, the routine experience is for the patient to note a lack of interaction, collaboration, or communication among specialists. This accords with the results of an EUFOREA patient advisory board, which noted a lack of coordination between physicians.^35^ As there is wide variability in the level of communication between respiratory and ENT specialists, the patient often acts as an intermediary; this is a key area where improvement is needed. Access to information from different specialists can be inconsistent; records are often not complete, and the way they are set up may also deter physicians from accessing them. However, even when information is accessible, it is not always utilised.

One advantage cited by the patient author for introducing the MDT approach is the potential positive impact of different ideas and methods for selecting the best treatment. From a patient perspective, it is encouraging to have more than one treatment option. However, when symptoms are severe (e.g. gasping for breath due to NPs), an MDT approach may be less important as the patient would be likely to prioritise the timely initiation of effective treatment, which may favour a single treating physician.

Overall, the MDT approach is considered important from the patient perspective. Most patients are treated by one physician (typically an ENT specialist via a referral of the pulmonologist after the severe asthma attack, in this patient's experience) rather than a team, and consequently may only receive ‘partial solutions’.

## 3 EXISTING MDT APPROACHES IN CRSwNP AND BARRIERS

### 3.1 Specialities in the MDT

Typical specialities involved in CRSwNP care include allergists/immunologists, ENT specialists, and pulmonologists.^4^, ^27^, ^36^ ENT specialists can help optimise management by first using nasal endoscopy to accurately diagnose the disease, assess its severity and subtype, and identify comorbid upper airway conditions; a detailed evaluation of the CT scans can then be performed to identify reasons for suboptimal outcomes.^4^ The inclusion of allergists/immunologists or pulmonologists in the MDT may be particularly important for patients with comorbid asthma to confirm asthma diagnosis, perform pulmonary function tests, and optimise asthma treatment. In some cases, patients with CRSwNP and asthma with comorbid otitis media with effusion (OME) may present with a dissociated response to treatment with biologics (i.e., improvement in one condition in tandem with worsening, or lack of improvement, of another);^37^ in these instances an MDT comprising expertise in both diseases is particularly important to determine the best course of treatment. The inclusion of pathologists, who can provide a detailed histopathologic analysis, is advantageous and may facilitate access to this information for other members of the MDT.^27^ A detailed histopathologic analysis can help determine the dominant cellular infiltrate and mucin eosinophil content, facilitating endotyping and thus informing the likely disease trajectory and best management strategy,^27^, ^38^, ^39^ for example, the benefit of surgery, the likelihood of recurrence post‐surgery, and the need for more intensive monitoring/management in those with histopathology indicative of refractory disease. Ideally, histopathologic analysis should be performed regularly (e.g. annually).

Primary care physicians (PCPs) are often the first, and regular, point of contact for a patient with CRSwNP.^40^ As the key point of contact with healthcare services for most people, PCPs play an important role in recognising and referring to people with CRSwNP symptoms. Additionally, once CRSwNP is diagnosed, PCPs should be aware when disease progression requires specialist referral. A statement from the EUFOREA patient advisory board highlighted that PCP education is needed to avoid delayed CRSwNP diagnosis/referral and incorrect medication use.^35^ Education may also address diagnostic delays by reducing the frequency of misdiagnoses. Evidence‐based position papers and other literature also help educate PCPs on the latest consensus towards diagnosis and treatment. For instance, the EPOS 2020 consensus paper reported that nasal congestion is the most common initial symptom of CRSwNP, and olfactory dysfunction, along with congestion, is the most specific symptom^41^; insights such as these help in timely diagnosis and specific treatment.

### 3.2 The role of the MDT in the CRSwNP patient journey

MDT plays a key role in four areas: diagnosis, comorbidity identification, optimising outcomes by combining surgical and non‐surgical approaches, and endotyping.^27^, ^42^ Patient‐centric disease management, good physician–patient communication, and shared decision‐making are all important in MDT approaches to ensure patient satisfaction with disease management strategy.^31^, ^43^ Adopting an MDT approach may facilitate shared decision‐making and a personalised treatment course, as patients under the care of an MDT may be presented with a wider range of treatment options.

Recurrence of NPs is a key issue for patients with CRSwNP who have undergone surgery, as it occurs in up to 60% of cases (median 20%) over 2 years of follow‐up.^44^ In such cases of persistent or recurring symptoms, an MDT approach is recommended.^45^ Having a range of clinical expertise within the care team may promote early detection of recurrence; for example, a pathologist might recognise increased eosinophils in a histopathology sample, whereas an ENT specialist may detect polyps by endoscopy. In addition, patients who have regular visits with their MDT to discuss their care plan may be more likely to be aware of the necessity of ongoing treatment adherence, even during periods when they feel their CRSwNP has improved.

**FIGURE 1:**
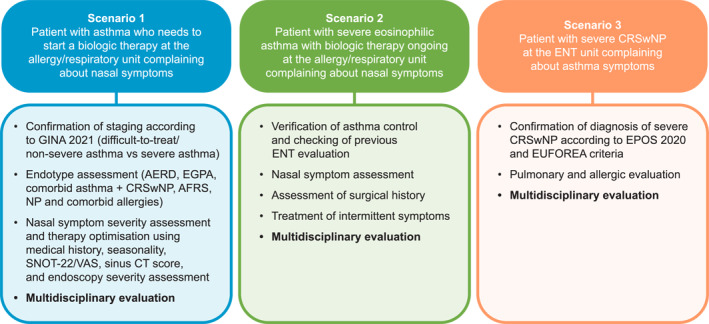
Patient scenarios illustrating where the MDT approach may be applied for patients with CRSwNP and comorbid severe asthma*. *Adapted from reference.^44^ AERD, aspirin‐exacerbated respiratory disease; AFRS, allergic fungal rhinosinusitis; CRSwNP, chronic rhinosinusitis with nasal polyps; CT, computed tomography; EGPA, eosinophilic granulomatosis with polyangiitis; ENT, ear, nose and throat; EPOS, european position paper on rhinosinusitis and nasal polyps; EUFOREA, european forum for research and education in allergy and airway diseases; GINA, global initiative for asthma; MDT, multidisciplinary team; NP, nasal polyps; SNOT‐22, sinonasal outcomes test; VAS, visual analogue scale.

Supported use of validated patient‐reported outcomes and eHealth tools can help patients monitor and manage their health and keep them informed.^31^ The role of the MDT during the patient journey is demonstrated by three different patient scenario management flows from Italy,^44^ highlighting how collaboration between pulmonologists, ENT specialists, and allergist/immunologists is important for patients with asthma reporting nasal symptoms, and vice versa (Figure 1). MDTs are already in the process of actively developing strategies to enhance their collaboration by examining combined treatment scenarios for patients with severe asthma and chronic rhinosinusitis with nasal polyps.^46^

### 3.3 Country‐specific differences in MDT approaches

A range of specialities are involved in CRSwNP management, depending on location; in literature originating from the US, the role of pathologists in the MDT is emphasised,^27^, ^42^ whereas in the Gulf region and Italy, the main specialities involved in MDTs are pulmonologists, allergist/immunologists, and ENT specialists.^30^, ^36^, ^44^ There may also be variability in the willingness to adopt virtual MDT meeting approaches where needed. For example, in the UK, a pilot study found a range of benefits to holding MDT meetings in a virtual forum (not specific to CRSwNP).^47^ This approach was highlighted as an effective and pragmatic alternative to in‐person meetings during the COVID‐19 pandemic as well as a potential standard component of future clinical workflows by a group of UK‐based HCPs.^48^ Another example is the recently published German guidelines on the ‘*treatment of CRSwNP with monoclonal antibodies*’, which encompass all relevant specialities and professional associations and provide evidence‐based recommendations that follow the principles of the Association of the Scientific Medical Societies in Germany (AWMF).^49^ It is also likely that the adoption of MDT approaches is variable both between and within countries, with some practices and HCPs adopting MDT practices, while others retain a more siloed approach with little collaboration between different specialities.

In contrast to asthma, there have been limited international initiatives to highlight the patient view and bring the impact of CRSwNP to the attention of health policymakers, the general public, and physicians.^35^

### 3.4 Barriers to adoption of MDT approaches in CRSwNP

The current standard of pathology review and clinicians' access to it represents a barrier to the successful adoption of an MDT approach. Differences in clinical approaches and working cultures between specialities may also present challenges; for example, ENT specialists may be more inclined towards a surgical approach and pulmonologists towards medical approaches.^50^ In some cases, there is also a lack of detailed understanding of other specialities, highlighting again the importance of physician education at both the primary and secondary care levels.^50^

Diagnosis and identification of comorbidities can also increase the complexity of patient care and must be considered by MDTs. A multidisciplinary diagnostic workup is recommended by the CRSwNP treatment guidelines to enable early and targeted interventions and to prevent disease worsening.^36^ However, barriers have been identified, such as in the routine assessment of olfaction, which may be due to differences between specialists in the use of University of Pennsylvania Smell Identification Test (UPSIT)/Sniffin sticks (more favoured by ENT specialists) and visual analogue scales (VAS) (preferred by allergist/immunologists).^36^ Patients with comorbid asthma and AERD more frequently require sinus surgery and have greater OCS use and disease recurrence,^51^, ^52^, ^53^ contributing to greater disease burden and impact on HRQoL.^10^ Additionally, patients with comorbid asthma and/or AERD may require treatment input from specialists in both upper and lower airway diseases. There may also be geographical differences in comorbidities that will affect the optimal MDT composition for the management of CRSwNP. For example, in Western countries, CRSwNP has typically been predominantly associated with type 2 inflammation and tissue eosinophilia, whereas Asian patients demonstrate a more mixed inflammatory endotype with type 1 and 3 inflammation even when type 2 inflammatory markers are also present; these differences tend to impact the occurrence of comorbidities in patients, particularly asthma.^54^, ^55^

Another potential barrier to the adoption of an MDT approach is the lack of consensus between HCPs from different specialities on the utility of certain aspects of CRSwNP endotyping and indicators of treatment responses for making treatment decisions. Endotyping might aid in the selection of (revision) sinus surgery, oral corticosteroid (OCS) treatment, and/or biological treatments.^7^, ^56^ Although a multidisciplinary panel reached consensus on the utility of eosinophils as a marker to endotype disease, consensus was not reached on the utility of immunoglobulin (Ig) E levels, potentially due to a lack of awareness of the EPOS 2020 guidelines^7^ among pulmonologists and allergists.^36^ Additionally, although an Italy based multidisciplinary panel reached consensus on 9/10 statements on disease severity and control, with nasal polyp score (NPS), Sino‐Nasal Outcome Test (SNOT)‐22 and OCS use considered important CRSwNP severity measures and treatment response indicators, the panel did not reach consensus on the use of Clinical‐Cytological Grading (CCG), which includes comorbidities, likely due to a lack of understanding around clinical cytologic grading among pulmonologists and allergists.^36^

While the MDT approach offers potential advantages for improving disease management, its effectiveness requires a high level of organisation and integration between multiple parties, which may be time consuming and challenging to achieve in practice. With this in mind, it is crucial to clearly define the role of each HCP in the collaborative process and their level of interaction.^57^ It is also necessary to ensure appropriate and continuous information exchange between MDT members and the patient to avoid any misunderstandings and delays in making treatment decisions.^57^ Tailored HCP education (i.e., for the nurse, clinician and specialist) is another key factor for success as it ensures appropriate patient identification, referral and treatment.^50^, ^57^ The MDT also needs to operate within the bounds of any limitations created by local policies and procedures.^50^

MDTs may have considerable implications for staffing and associated costs,^50^ given the breadth of HCP involvement and the need to include multiple specialists in the discussions. However, this may be far outweighed by the potential to improve efficiencies of care that may reduce the cost of patient management overall. It has been shown that indirect costs, including missed workdays and absenteeism, are a major component of CRSwNP disease burden.^58^ In the US, the indirect costs (∼20 billion USD) were estimated to be substantially more than the direct costs (∼6–13 billion USD), with similar findings being reported from the EU and other geographies.^58^ Therefore, additional direct spending on healthcare (e.g., in creating and pursuing the MDT approach) may be justified if it can help control indirect costs and overall socioeconomic burden of disease.

## 4 LESSONS LEARNT FROM ALLIED AND RARE DISEASES

MDT approaches are recommended for a range of diseases, including allergic rhinitis, diabetes, and rheumatoid arthritis.^57^, ^59^, ^60^ The benefits of an MDT approach are exemplified by aspirin desensitisation in highly refractory patients with AERD. In these patients, endoscopic sinus surgery performed by an ENT specialist may decrease the severity of aspirin‐induced reactions during aspirin desensitisation, which is usually carried out by an allergist/immunologist.^61^, ^62^

In addition, the MDT approach has benefitted patients with eosinophilic granulomatosis with polyangiitis (EGPA) and hypereosinophilic syndrome (HES); due to the rarity and heterogenous presentation of these diseases across multiple organ systems, MDT evaluation and management is recommended.^63^, ^64^, ^65^ Guidelines on the use of MDTs for HES emphasise the need to prioritise expert opinion on a case‐by‐case basis over following consensus statements and guidelines prescriptively, owing to the wide range of clinical conditions grouped under the term.^66^

Additionally, the management of severe asthma has benefited from an MDT approach, which has been shown to reduce corticosteroid exposure, exacerbation rates, and hospitalisations, as well as improve patient experience.^33^ Diagnosing severe asthma is a complex process; the use of a one‐day multidisciplinary assessment at dedicated asthma centres in France, aimed at confirming the diagnosis and establishing a management strategy, has proven successful at mitigating this and optimising asthma control.^32^ In some cases, this is achieved without a step‐up in asthma treatments, possibly due to improved patient education.^32^ The Newcastle model has been developed in Newcastle, Australia, as a person‐centred model of care used by an MDT to develop an individualised treatment plan for asthma. Initial assessment involves clinical review by team members, synthesis of relevant data, and development of a diagnosis and management plan. Monthly MDT meetings are held to review and discuss difficult cases, with each speciality having input into assessment and recommendations. In addition to providing recommendations for specific cases, this process has educational benefits for all MDT members.^34^ Key members of the airway disease MDT in this model are consultant physicians, physicians‐in‐training, specialist and consultant respiratory nurses, speech pathologists, dietitians, psychologists, physiotherapists, respiratory scientists, and pharmacists. The model emphasises that not every speciality has to be involved in the day‐to‐day management but that access should be available if needed.^34^

Access and multidisciplinary working can also be improved by digital and virtual approaches, which allow communication between specialists spread across different sites and in situations where physical meeting is not convenient or not possible, for example, during the COVID‐19 pandemic.^48^ Allergists and other HCPs in the allergy/associated airway disease field had a high level of patient contact during the COVID‐19 pandemic^67^; thus, virtual approaches to MDT management may be particularly relevant when these specialities form a key component. A previous study found that virtual MDTs are valued by clinicians and patients and can improve diagnosis, treatment, and discharge planning, and could also facilitate coordinated care for patients with additional mental health needs.^47^

## 5 ADDRESSING UNDER‐RECOGNISED ASPECTS OF CRSwNP FOR MDTS

Under‐recognised aspects of CRSwNP include its impact on overall physical and mental health, social functioning, work productivity, ability to exercise, and sleep.^9^, ^68^ An often underappreciated facet of the experience of CRSwNP is that even short‐course OCS use is associated with its own burden. In children, this can include vomiting, behavioural changes, sleep disturbances, and infection; in adults, a dose–response relationship has been observed between cumulative OCS exposure and adverse events, while short‐term use is cumulatively associated with osteoporosis, hyperglycaemia, and muscle weakness.^69^ Further, the development of OME, which can lead to hearing loss, occurs in 25% of patients with CRSwNP but is under‐diagnosed due to a lack of knowledge about ear disease by non‐ENT specialists.^37^ Therefore, there is a need to consider other individuals in the MDT, both specialists (e.g. PCPs, allied health practitioners, pulmonologists/sleep specialists, endocrinologists, rheumatologists) and ‘non‐traditional’ MDT members (e.g. psychosocial specialists, professional societies, patient organisations/patient advocacy groups [PAGs]). Additionally, there may be an under‐recognised need for MDTs that combine different specialities within one clinic to allow specialists to share their expertise in real time and align on an optimal care plan.

As highlighted in the patient experience section, appropriate access to medical records between different physicians is an issue that may be overlooked; this may be in part ameliorated by the use of MDTs, although wider reform and investment may be required to optimise their use. Additionally, there is a need for wider recognition of the fact that patient HRQoL does not always correlate well with objective measures of NP burden.^6^ HCP education would go some way to addressing this; however, an MDT approach may also help with gauging patient disease burden on an individualised basis owing to the presence of a wider range of experience.

Although a systematic review of the available literature was beyond the scope of this paper, the evidence discussed here, and insights from the patient case study, suggest that most patients with CRSwNP would be likely to benefit from collaborative MDT management of their disease. However, there are evidence gaps, and areas for additional research might include a systematic review of the primary literature on the potential impact of comorbidities on the composition of the ideal MDT the benefits of MDTs for patients with inadequately controlled CRSwNP who are receiving or will require biological treatment (e.g., in comparison to those managed with OCS or surgical intervention), and the effects of coordinated HCP care above and beyond traditional clinical outcomes (e.g., exploring factors that could mitigate the cost impact of MDT and considering how MDT might impact the socioeconomic burden of CRSwNP, as well as HRQoL, medication use or need for surgery). Prospective, real‐world interventional studies are required to fully appreciate the impact of MDTs on patient outcomes in CRSwNP, and to explore how the implementation of such care models in different healthcare settings may help to optimise and individualise patient care from the point of diagnosis through treatment and follow‐up.

## 6 KEY CONSIDERATIONS WHEN DEVELOPING AN MDT APPROACH

**FIGURE 2:**
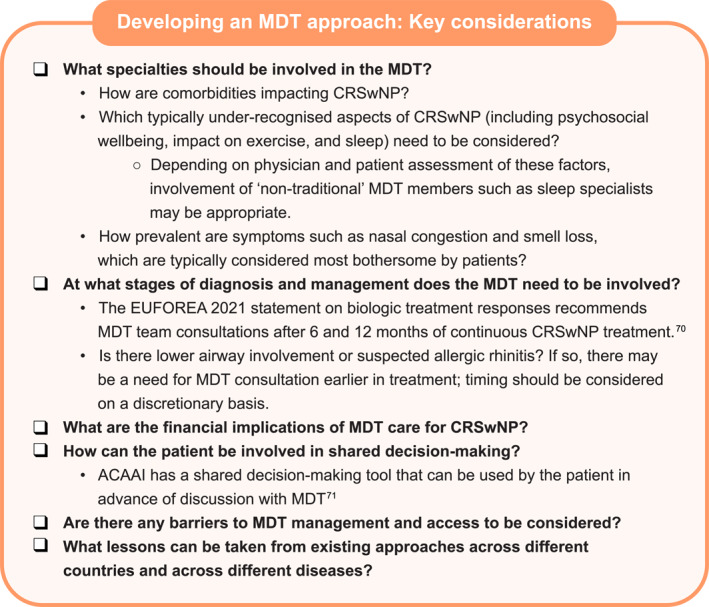
Key considerations when developing an MDT approach.^70^, ^71^ ACAAI, american association of allergy, asthma and immunology; CRSwNP, chronic rhinosinusitis with nasal polyps; EUFOREA, european forum for research and education in allergy and airway diseases; MDT, multidisciplinary team.

Key considerations when developing an MDT approach for individual patients are summarised in Figure 2.

## 7 CONCLUSIONS

Overall, this review of the literature on the use of the MDT approach in CRSwNP found that MDTs may be a beneficial tool for reducing diagnostic delays and providing a fuller range of treatment options to patients commensurate with well‐established medical and surgical best practices targeting this complex disease burden. They were supported by the personal experience of a patient author who highlighted a number of potential benefits from a patient perspective, including increasing consistency in access to and use of electronic health records and reducing the burden on patients, who may find themselves visiting several speciality physicians separately and acting as an intermediary between them.

Despite barriers to the adoption of an MDT approach, cross‐functional collaboration within such spaces is a valuable tool for ensuring consistency of care as the scientific knowledge of CRSwNP evolves, by encouraging discussion and knowledge sharing between specialities. As patient experience and knowledge of the disease increased, the quality of their communication with physicians improved, highlighting the need for ongoing patient education to facilitate patient participation in shared decision‐making alongside the MDT.

There is a current lack of robust methodology to quantify the success of an MDT approach; this is an area that would benefit from future research. Nonetheless, a benefit to patients has been demonstrated in severe asthma patients in several countries across a range of healthcare systems. To provide more uniform patient care in CRSwNP, specific guidance on the importance of MDTs and practical advice on developing an MDT approach from organisations such as International Consensus in Allergy and Rhinology (ICAR), EPOS and key professional societies are needed.

## AUTHOR CONTRIBUTIONS

Oliver Pfaar, Anju T. Peters, Camille Taillé, Jared Silver, Robert Chan and Peter W. Hellings contributed to conception of this review. All authors contributed to interpretation of data for this review article in addition to critically reviewing it for important intellectual content and providing final approval of the version to be published. Jared Silver contributed to data collection.

## CONFLICT OF INTEREST STATEMENT

OP reports grants and/or personal fees and/or travel support from ALK‐Abelló, Almirall S.A., Allergopharma, Stallergenes Greer, HAL Allergy Holding B.V./HAL Allergie GmbH, Bencard Allergie GmbH/Allergy Therapeutics, Lofarma, ASIT Biotech Tools S.A., Laboratorios LETI/LETI Pharma, GlaxoSmithKline, ROXALL Medizin, Novartis, Sanofi‐Aventis und Sanofi‐Genzyme, Med Update Europe GmbH, streamedup! GmbH, Pohl‐Boskamp, Inmunotek S.L., John Wiley and Sons, AS, Paul‐Martini‐Stiftung (PMS), Regeneron Pharmaceuticals Inc., RG Aerztefortbildung, Institut für Disease Management, Springer GmbH, AstraZeneca, IQVIA Commercial, Ingress Health, Wort&Bild Verlag, Verlag ME, Procter&Gamble, ALTAMIRA, Meinhardt Congress GmbH, Deutsche Forschungsgemeinschaft, Thieme, Deutsche AllergieLiga e.V., AeDA, Alfried‐Krupp Krankenhaus, Red Maple Trials Inc., Königlich Dänisches Generalkonsulat, Medizinische Hochschule Hannover, ECM Expro&Conference Management, Technische Universität Dresden, Lilly, Paul Ehrlich Institut (PEI), Japanese Society of Allergy, Forum für Medizinische Fortbildung, Dustri Verlag, all outside the submitted work and within the last 36 months; and he is Vice President of EAACI and member of EAACI Excom, member of ext. board of directors DGAKI; coordinator, main or co‐author of different position papers and guidelines in rhinology, allergology and allergen‐immunotherapy and Associate Editor of the journal(s) *Allergy* and *Clinical Translational Allergy (CTA)*. ATP has received research grants from Sanofi Regeneron, AstraZeneca, and Merck, and has participated in advisory boards for Sanofi Regeneron, AstraZeneca, GSK, Chiesi, Eli Lilly and Merck. CT has received lecture or advisory board fees and grants from AstraZeneca, Sanofi, GSK, Chiesi, Stallergenes Greer and Novartis. TT has no conflicts of interest to declare. JS was a former employee of GSK at the time of manuscript development and holds financial equities in GSK. JS is currently an employee of Amgen, and holds financial equities in Amgen. RC is an employee of GSK and holds financial equities in GSK. PWH reports research grants and/or lecture fees by GSK, Sanofi/Regeneron, Viatris, Stallergenes and Novartis.

## Data Availability

Data sharing is not applicable to this article as no datasets were generated or analysed during the current study.
